# MicroRNA-495 inhibits proliferation of glioblastoma multiforme cells by downregulating cyclin-dependent kinase 6

**DOI:** 10.1186/1477-7819-11-87

**Published:** 2013-04-17

**Authors:** Shu-Mei Chen, Hua-Chien Chen, Shu-Jen Chen, Chiung-Yin Huang, Pin-Yuan Chen, Tai-Wei Erich Wu, Ly-Ying Feng, Hong-Chieh Tsai, Tai-Ngar Lui, Chuen Hsueh, Kuo-Chen Wei

**Affiliations:** 1 Graduate Institute of Clinical Medical Sciences, College of Medicine, Chang Gung University, No.259 Wen-Hwa 1st Rd, Kweishan 333, Taoyuan, Taiwan; 2 Department of Neurosurgery, Taipei Medical University-Wan Fang Hospital, Taipei Medical University, No.111, Sec. 3, Xinglong Rd, Taipei 116, Taiwan; 3 Molecular Medicine Research Center and Graduate School of Basic Medical Science, Chang Gung University, No.259 Wen-Hwa 1st Rd, Kweishan, Taoyuan 333, Taiwan; 4 Department of Neurosurgery, Chang Gung Memorial Hospital at Linkou, Chang Gung University, No.5 Fu-Shin Street, Kweishan, Taoyuan 333, Taiwan; 5 Department of Pathology, Chang Gung Memorial Hospital at Linkou, Chang Gung University, No.5 Fu-Shin Street, KweishanTaoyuan 333, Taiwan

**Keywords:** CDK6, Glioblastoma multiforme, MicroRNA-495, Tumor progression

## Abstract

**Background:**

Glioblastoma multiforme (GBM) is the most aggressive type of glioma and carries the poorest chances of survival. There is therefore an urgent need to understand the mechanisms of glioma tumorigenesis and develop or improve therapeutics. The aim of this study was to assess the possible prognostic value of cyclin-dependent kinase 6 (CDK6) and the effects of microRNA-495 (miR-495) manipulation on CDK6 expression and cell survival in glioma cells.

**Methods:**

Analyses of clinical specimens from GBM patients were used. Expression of CDK6 was analyzed by real-time polymerase chain reaction (RT-PCR), Western blotting, and immunohistochemistry. Expression of CDK6 was also analyzed after over-expression of miR-495 in T98 cells; both cell proliferation and RB phosphorylation were examined. Cell proliferation, cell cycle distribution, and RB phosphorylation were also examined after knockdown of CDK6 in U87-MG and T98 cells.

**Results:**

Analyses of clinical specimens from GBM patients identified that CDK6 is significantly expressed in gliomas. CDK6 antigen expression was higher in tumor cores and margins than in adjacent normal brain tissues, and higher levels of CDK6 expression in the tumor margin correlated with decreased survival. Over-expression of miR-495 in T98 cells downregulated the expression of CDK6 and inhibited retinoblastoma phosphorylation, and knockdown of CDK6 in U87-MG and T98 cells by siRNAs resulted in cell cycle arrest at the G1/S transition and inhibition of cell proliferation.

**Conclusions:**

This study revealed miR-495 is down-regulated in glioma tissues. Furthermore, miR-495 regulated CDK6 expression and involved in glioma cell growth inhibition, which indicated the possible role of miR-495 in tumor progression.

## Background

Malignant gliomas, the most common primary brain malignant tumor, are aggressive, highly invasive, and neurologically destructive human cancers. Glioblastoma multiforme (GBM), the most aggressive manifestation of gliomas, typically affects adults between 45 and 60 years of age. Despite advances in surgical techniques, radiotherapy, and chemotherapy, the prognoses of patients with GBMs remain dismal [1], largely because of recurrences from extensions of tumor cells to adjacent regions.

Establishing the molecular basis of the tumorigenesis of malignant gliomas is crucial to improving current therapies and developing new ones. It has been suggested that gene expression profiles from glioma specimens might predict patient outcome more accurately than pathological criteria [2,3]. In particular, the cell cycle regulator enzyme cyclin-dependent kinase 6 (CDK6) is over-expressed in GBM [4,5], although not all the over-expression can be explained by genomic amplification, suggesting that other gene regulatory mechanisms might be involved [5].

MicroRNAs (miRs) are small (18 to 22 nucleotides), non-coding RNAs with post-transcriptional gene silencing activity [6,7] that regulate gene expression by associating with the 3^′^-untranslated region (3^′^-UTR) of genes and inhibiting protein translation [8]. miRs can also destabilize and mediate the degradation of RNA transcripts [9]. In addition to their role in normal development, miRs are also associated with carcinogenesis [10,11]. For example, miR-21 is over-expressed in glioblastoma, whereas its inhibition inhibits glioblastoma growth *in vitro* and *in vivo*[12]. In contrast, some miRs act as tumor suppressors: miR-34 functions as a p53-dependent tumor suppressor in neuroblastoma [13], and miR-124 inhibits *CDK6* and thus reduces tumor proliferation in medulloblastoma [14].

In the present study, we reveal that miR-495 is significantly decreased in GBM samples, and sequence analysis using TargetScan 6.2 identified the 3^′^-UTR of *CDK6* as a potential target of miR-495. The present study also demonstrates that expression of CDK6 antigen, particularly in the tumor margins, is prognostic of poor patient survival. Furthermore, *CDK6* is downregulated by over-expression of miR-495 in GBM cells, suggesting that miR-495 might play an important role in malignant glioma tumorigenesis.

## Methods

### Patient population

The patient population consisted of 20 adults (16 male, 4 female; mean age at sampling = 56.5 yrs). Written, informed consent was obtained from all patients, and the study was approved by, and performed according to, the guidelines of the Institutional Review Board of Chang Gung Memorial Hospital (Approval #94-182 and #98-0341B). GBM was verified in histological specimens between Feb 2004 and July 2009 by a neuropathologist according to World Health Organization criteria. All 20 cases were classified as grade 4, with 18 cases of GBM and 2 cases of glioblastoma with oligodendroglioma.

### Region-specific specimen collection

Deep-seated tumors were removed using an intraoperative navigation system (BrainLAB, Feldkirchen, Germany) that minimized invasiveness and maximized patient safety and accurate tumor resection. Brain tissue samples were collected from the resection zone, categorized as peripheral normal brain, tumor marginal tissue or tumor core, and stored in liquid nitrogen as described previously [15].

### Real-time polymerase chain reaction

The following primers and probe for *CDK6* were used: forward: 5^′^-TGCACAGTGTCACGAACAGA-3^′^; reverse: 5^′^-ACCTCGGAGAAGCTGAAACA-3^′^ (Mission Biotech, Taipei, Taiwan); probe: 5^′^-CATATTGCTTCAATGCTTCAGCTCCACCTG-3^′^ (Applied Biosystems, Carlsbad, CA, USA). RT-PCR was performed as follows: 50°C for 2 min; 95°C for 15 min; 40 cycles of 95°C for 15 s and 60°C for 1 min. Experiments were performed in triplicate. Gene expression levels were calculated by the ΔΔC_t_ method and normalized against the RPL35A control. Expression of *hsa-miR-495* was analyzed using specific primers and TaqMan probe according to the manufacturer’s protocol (Applied Biosystems) and normalized in each sample against expression of the *RNU44* gene.

### Immunoblotting

Brain tumor samples were washed twice with ice-cold phosphate-buffered saline (PBS) and lysed on ice in chilled T-PER tissue protein extraction reagent (Pierce, Rockford, IL, USA) containing a protease inhibitor cocktail (Sigma-Aldrich, St. Louis, MO, USA). Lysates were cleared by centrifugation, and total protein concentrations were determined by Bradford assay (Bio-Rad, Hercules, CA, USA). Protein samples (30 μg/lane) were separated on 12% polyacrylamide gels by sodium dodecyl sulfate/polyacrylamide gel electrophoresis and transferred to polyvinylidene difluoride membranes (Millipore, Billerica, MA, USA). Blots were blocked overnight in 20 mM Tris–HCl, 150 mM NaCl, 0.1% Tween-20, 0.5 μM EDTA (pH 7.4) containing 5% non-fat dry milk, incubated for 2 h with anti-human CDK6 antibodies (1:1,000; Santa Cruz Biotechnology, Santa Cruz, CA, USA) or anti-human phosphorylated retinoblastoma (pRB) antibodies (1:500; Santa Cruz Biotechnology), and then incubated with horseradish peroxidase-conjugated goat anti-mouse or anti-rabbit IgG (1:10,000; PerkinElmer, Waltham, MA, USA) for 1 h. Specific labeling was visualized using the Western Lightning Detection kit (PerkinElmer) according to the manufacturer’s instructions.

### Immunohistochemistry

Tissue sections from peripheral, marginal, and tumor core regions were deparaffinized, treated with 3% H_2_O_2_ for 10 min at room temperature, and then microwaved in 0.01 M citrate buffer (pH 6.0) to retrieve antigenicity. The sections were blocked with 1% bovine serum albumin in PBS for 20 min at room temperature and incubated overnight with a mouse anti-human CDK6 monoclonal antibody (Santa Cruz Biotechnology) diluted 1:100 in the blocking buffer. Samples were washed four times with PBS and incubated with goat anti-mouse IgG (PerkinElmer). Immunocomplexes were visualized by an LSAB 2 HRP kit (Dako, Carpinteria, CA, USA) using 3,3^′^-diaminobenzidine tetrachloride as a substrate. Sections were counterstained lightly with hematoxylin, dehydrated with a graded alcohol series, cleared with xylene, and mounted with coverslips.

### Cell lines

Two malignant glioma cell lines (T98 and U87-MG) were purchased from American Type Culture Collection. T98 and U87-MG cells were cultured in modified Eagle’s medium (MEM, Life Technologies, Grand Island, NY, USA) supplemented with 10% fetal bovine serum (Life Technologies), 1% non-essential amino acids, and 1% penicillin and streptomycin (Life Technologies).

### siRNA transfection

The siRNAs for *CDK6* (43900824 ID: S51 and S53) and control siRNA#1 were purchased from Ambion (Austin, TX, USA). T98 and U87-MG cells were transfected with siRNA using Lipofectamine 2000 (Invitrogen, Carlsbad, CA, USA) according to the manufacturer’s protocol.

### miRNA transfection

Over-expression of miR-495 was accomplished using BLOCK-iT™ Pol II miR RNAi Expression Vector Kits (Invitrogen) to generate an expression clone containing a double-stranded oligo encoding a pre-miR-495 sequence. The constructs were transfected into T98 cells using Lipofectamine 2000 (Invitrogen) according to the manufacturer’s protocol.

### Cell cycle analysis

T98 and/or U87-MG cells were harvested from medium following siRNA treatments. Cells were fixed in 70% ethanol and stored at −20°C until use. After thawing and equilibrating to room temperature, the cells were resuspended in PBS containing 0.5% Triton X-100 and 0.05% RNase, stained with 50 μg/mL propidium iodide (Sigma), and maintained at 4°C for 30 min. Cell cycles were analyzed by flow cytometry using a FACS-Calibur incorporating Cell Quest software (BD Biosciences, Mountain View, CA, USA) and WinMDI 2.8 software (Salk Institute for Biological Studies, La Jolla, CA, USA).

### MTT assay

A total of 1 × 10^4^ T98 or U87-MG cells were cultured in 96-well tissue-culture plates overnight, and then treated with siRNA or miRNA as described above. After 1 to 5 days, glioma cells were incubated for three hours in 100 μL of 0.1-mg/mL 3-(4,5-dimethylthiazol-2-yl)-2,5-diphenyltetrazolium bromide (MTT; Sigma). Cells were resuspended in 200 μL isopropanol (to dissolve the formazan), and the optical density of the solution was determined using a spectrometer at an incident wavelength of 570 nm. Cell viabilities in experimental wells were expressed as a percentage of the viability in the control well.

### Statistical analyses

Wilcoxon signed-rank tests were used to compare the levels of *CDK6* and miR-495 expression between brain tumor core, margin, and normal peripheral tissues. Survival curves were estimated using the Kaplan-Meier method, with progression-free survival defined as the period from initial diagnosis to tumor progression (or the last follow-up). Logrank tests were used to evaluate the association between expression levels in different tumor sections and patient survival; GBM cases were categorized as having high or low expression levels relative to the median values. All analyses were performed using SAS statistical software version 9.1.3 (SAS Institute Inc., Cary, NC, USA). All *P* values were two-sided, with 0.05 being considered significant.

## Results

### Expression of CDK6 in human glioma tissues

**Figure 1:**
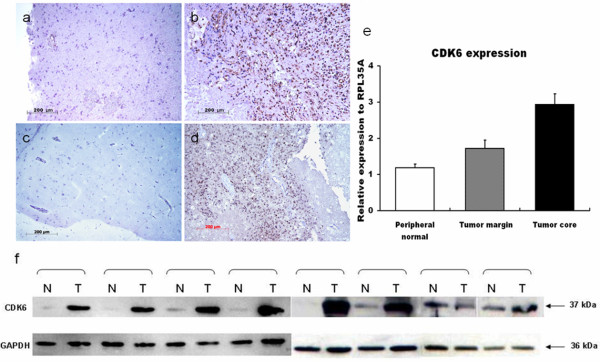
**CDK6 expression in surgically defined regions.** Immunohistochemical staining of normal peripheral tissues (**a,c**) and glioma tissues (**b,d**); (**e**) RT-PCR analysis of *CDK6* expression in samples from different regions of gliomas; (**f**) CDK6 protein expression detected by immunoblotting. N: Normal peripheral tissue; T: Tumor tissue.

In 20 sets of tissue samples collected from different brain regions, expression of the *CDK6* gene was upregulated significantly in tumor core samples (*P* = 0.0042) relative to peripheral normal brain, although expression decreased progressively in samples taken more distally (Figure 1e). Immunoblotting confirmed increased CDK6 protein expression in malignant gliomas consistent with the elevated transcript levels (Figure 1f), and immunohistochemistry verified the graded distribution of the protein in the tumors (Figure 1a–d).

### Correlation of CDK6 expression with survival rates

**Figure 2:**
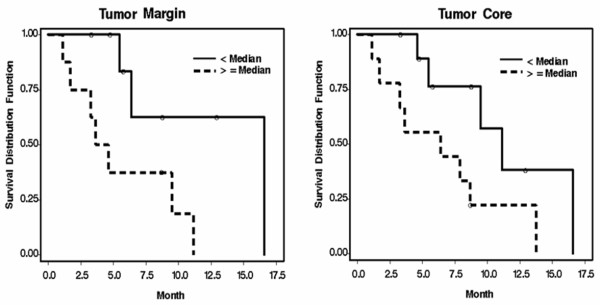
**Kaplan-Meier survival curves of 20 GBM patients.** Survival time is defined as the time from diagnosis to progression or last known follow-up, where circles represent censored values. Solid lines represent low levels of *CDK6* expression (*i.e.*, less than the median value); dotted lines indicate cases with *CDK6* expression equal to or above the median value.

Kaplan-Meier survival plots showed a relationship between *CDK6* expression and survival rates in GBM patients. However, although higher *CDK6* transcript levels in tumor cores indicated poorer survival, the correlation was not statistically significant (Figure 2; *P* = 0.0529). In contrast, patients with higher levels of *CDK6* transcripts in the tumor margin had significantly poorer prognoses than those with submedian levels of *CDK6* (Figure 2; *P* = 0.0279).

### Knockdown of *CDK6* inhibits cell proliferation and arrests glioma cells

**Figure 3:**
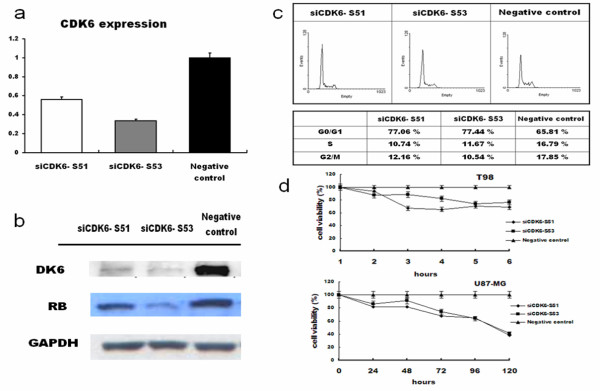
**Effects of *CDK6 *knockdown by siRNAs.** (**a**) RT-PCR analysis of *CDK6* expression in *CDK6*-knocked down cells; (**b**) Immunoblot analysis of CDK6 and pRB protein; (**c**) DNA flow cytometry on cell cycle stage distribution in T98 cells harvested 48 h after transfection; (**d**) Viability of *CDK6*-knocked down cells.

Knockdown of *CDK6* reduced expression of both *CDK6* transcripts (Figure 3a) and protein (Figure 3b). Furthermore, levels of pRB (pSer 795), a known target of CDK6, were also reduced in response to *CDK6* knockdown (Figure 3b). Furthermore, knockdown of *CDK6* inhibited proliferation of T98 and U87-MG cells (Figure 3d). Cell cycle analyses by flow cytometry showed that T98 and U87-MG cells transfected with siCDK6 had a higher percentage of cells in G_0_/G_1_ phase than negative control cells (Figure 3c). Concomitantly, the percentage of cells in S phase and in G_2_/M phase was reduced, suggesting that silencing CDK6 inhibits growth by arresting the cell cycle at the G_1_/S transition.

### miR-495 is downregulated in GBM tissues

**Figure 4:**
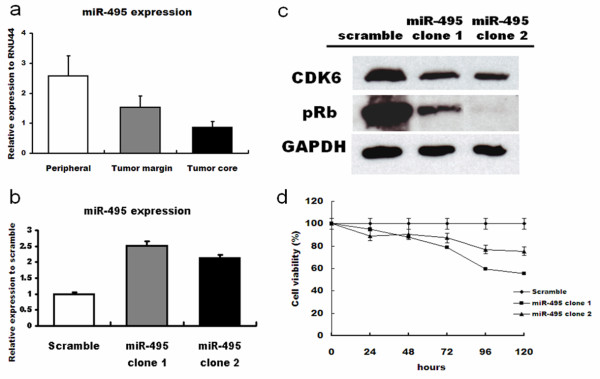
**Expression of miR-495 in glioma cells and tissues.** (**a**) miR-495 expression in tissue samples from defined regions of glioma patients; (**b**) miR-495 expression in T98 cells transfected with miR-495; (**c**) Immunoblot analyses of CDK6 and pRB protein expression in T98 cells over-expressing miR-495; (**d**) MTT assay of effects of miR-495 over-expression in T98 cells.

RT-PCR analysis was used to measure expression of mature miR-495 sequences in the three defined regions from patients with GBM. Expression of miR-495 was decreased in both the tumor core and tumor margin relative to normal peripheral brain tissue, although the downregulation was more pronounced in the tumor core (Figure 4a; *P* = 0.0004).

### miR-495 inhibits CDK6 expression, pRB levels, and inhibits growth of GBM cells

Transfection of miR-495 into T98 cells reduced expression of *CDK6* protein (Figure 4c). Furthermore, pRB levels were also reduced in response to miR-495 transfection (Figure 4c). MTT analyses confirmed that over-expression of miR-495 inhibited the growth of T98 cells (Figure 4d).

## Discussion

Establishing the factors responsible for the development, proliferation, metastasis, and recurrence of brain tumors could help identify potential diagnostic markers or targets for new therapeutic regimens. In the present study, we report the potential role of CDK6 and its possible regulatory mechanism in glioblastoma. CDK6 is a member of a family of serine/threonine kinases involved in the control of cell cycle progression, interacting with cyclin D to phosphorylate the retinoblastoma protein during the G_1_/S transition [16]. Alterations of CDK6 expression have been reported in several tumor types, including T-cell lymphoma [17], medulloblastoma [18], neuroblastoma [19], and gastric cancer [20]. Elevated expression of CDK6 protein has also been reported in GBM, and CDK6 is thought to be associated with astrocytic tumorigenesis [4,5]. The present study indicates that CDK6 is expressed both transcriptionally and translationally at higher levels in GBM tissues. Furthermore, CDK6 is expressed more abundantly in the tumor core than in marginal or peripheral regions. Thus, CDK6 expression correlates with the density of malignant cells in tumor-affected regions, and may be involved in the microenvironments of scattered glioma cells.

Analysis of survival data from patients with GBM reveals that, although elevated expression of CDK6 RNA in the tumor cores trended toward poor survivability, the results were not statistically significant. In contrast, the correlation between *CDK6* expression in tumor marginal regions and poor survival was significant, possibly because tumor recurrence usually originates in the residual tumor margin after tumor resection. Thus, even tissues surrounding a tumor can provide valuable prognostic indicators for patient survival. Since only deep seated glioma would be resected under the help of an intraoperative navigation system, collection of sample sets including tumor core, margin, and periphery is strictly limited. However, these restricted samples still provide statistically important information, which indicate the over-expression of CDK6 in the tumor margin may correlate with poor prognosis.

In GBM, CDK6 is amplified genomically in some, but not all, of the tumors that over-express CDK6 protein, suggesting that other regulatory mechanisms might control CDK6 expression in GBM tumors [5]. miRNAs are small non-coding RNA molecules that regulate protein expression by cleaving or repressing the translation of target mRNAs. Aberrant expression of miRNAs in cancer is well documented, *e.g.*, in chronic lymphocytic leukemia [21], breast carcinoma [22,23], lung cancer [24], papillary thyroid carcinoma [25], colon carcinoma [26], pancreatic tumors [27,28], and primary glioblastoma [29,30]. These miRNAs regulate the expression of signaling molecules such as cytokines, growth factors, transcription factors, and pro- and anti-apoptotic genes. miRNAs also act as regulators of many neuronal functions [31]. Specifically, miR-125b regulates the proliferation of U251 glioma stem cells through the cell cycle-regulated proteins CDK6 and CDC25A [32]. Similarly, miR-124 and miR-137 inhibit proliferation of GBM cells and induce differentiation of brain tumor stem cells by inhibiting CDK6 [33].

miR-495 regulates the expression of brain-derived neurotrophic factor in brain [34] and hepatocyte nuclear factor 6 and Onecut 2 in developing liver and pancreas [35]. miR-495 also inhibits the migration and invasion of human gastric cancer cells by directly interacting with PRL-3 [36]. In this study, we found that miR-495 was expressed at low levels in malignant glioma tumor tissues, therefore suggesting that its up-regulation can lead to the growth inhibition of glioma cells. Furthermore, TargetScan 6.2 predicts six potential target sites of miR-495 in the 3^′^-UTR of *CDK6*. The present work confirms miR-495 as a regulator of CDK6 expression. miR-495 was expressed at lower levels in malignant glioma tumor tissues than in normal peripheral tissues, and its upregulation inhibited the growth of glioma cells *in vitro*. Further studies will be required to determine the precise mechanism by which over-expression of miR-495 decreases CDK6 expression and inhibits glioma cell proliferation.

Given that aberrantly expressed miRNAs can play key roles in the development of human cancers, recent studies increasingly emphasize the potential therapeutic applications of these molecules [37-39]. Both loss and gain of miRNA function can result in cancer development through the upregulation and silencing, respectively, of particular target genes. Correcting these miRNA dysregulations by either miRNA antagonists or miRNA mimics might represent a useful strategy to interfere therapeutically with key pathways involved in cancer development.

## Conclusions

In summary, high levels of expression of the cell cycle regulatory protein CDK6 are indicative of a poor prognosis in patients with GBM, especially when expressed in tumor margins. In contrast, expression of miR-495 (a putative regulator of CDK6 expression) is low in glioma tumor tissues, whereas its over-expression appears to block or delay the G_1_/S transition of the cell cycle by downregulating CDK6, thus inhibiting proliferation of malignant glioma cells. In the present study, we report the significance of analyzing tissues adjacent to glioblastoma tumors, and the correlation of CDK6 levels and better survival. Since tumor adjacent tissues of glioblastomas are the therapeutic target area of post-operational treatment, the biological nature of tumor adjacent regions may be the key factor that affects the prognosis of glioblastoma. The efforts on investigating the biological nature of this area may provide important information for therapeutic strategy selection and development.

## Abbreviations

CDK6: Cyclin-dependent kinase 6; GBM: Glioblastoma multiforme; miR-495: MicroRNA-495; miRNA: microRNA; PBS: Phosphate-buffered saline; pRB: Phosphorylated retinoblastoma; RT-PCR: Real-time polymerase chain reaction; UTR: Untranslated region

## Competing interests

The authors declare that they have no competing interest.

## Authors’ contributions

SMC conducted the experiments, searched literature and drafted the manuscript; HCC and SJC participated in its design and coordination; CYH, LYF, PYC, TWW, HCT, and TNL assisted during the experiments and in the manuscript preparation; CH carried out the tissue preparation, (immuno)histology and edited the manuscript for its scientific content; KCW was responsible for the operations, follow-up of the patients and data preparation, and conceived the study. All authors have read and approved the final manuscript.
